# 3D Geometry-Aware Efficient Feature Matching for Weakly Textured Scenes

**DOI:** 10.3390/jimaging12060253

**Published:** 2026-06-07

**Authors:** Libo Sun, Yidong Yan, Wenqi Yang, Wenhu Qin

**Affiliations:** School of Instrument Science and Engineering, Southeast University, Nanjing 210096, China; 220233637@seu.edu.cn (Y.Y.); 220253803@seu.edu.cn (W.Y.)

**Keywords:** feature matching, weak texture, kinematics supervision, RGB-D geometry, industrial vision, edge computing

## Abstract

Local feature matching plays a critical role in robotic SLAM and visual localization. However, in weakly textured indoor industrial environments, lightweight appearance-based methods often struggle to learn discriminative and stable local features. To address this challenge, this paper proposes GAEFeat, short for Geometry-Aware Efficient Feature, a lightweight vision–geometric feature learning network. To address the scarcity of specialized training data, we integrated robotic arm pose priors with depth information to automatically generate cross-view supervision signals and surface-normal labels. Based on this strategy, we constructed two complementary datasets, including a simulated dataset and a real-world dataset, to support feature learning and evaluation in weakly textured indoor industrial environments. For feature extraction, we design a dual enhancement mechanism consisting of a geometric auxiliary branch and a geometry-aware enhancement (GAE) module. The former guides the network to perceive local surface structures through surface normal supervision, while the latter utilizes a gating mechanism to achieve deep fusion between geometric priors and 2D texture descriptors. Experimental results demonstrate that GAEFeat achieves strong robustness and high inference efficiency in relative pose estimation, homography estimation, and visual localization tasks, with particularly notable advantages in near-field, weakly textured industrial scenes. The framework achieves an inference latency of only 3.9 ms on the NVIDIA Jetson AGX Orin edge platform, demonstrating its real-time capability and practical potential for deployment in edge computing environments.

## 1. Introduction

With the rapid advancement of embodied intelligence, robots are increasingly deployed in complex, unstructured environments, posing greater demands on visual perception. As a fundamental component of robotic vision systems, visual feature matching establishes local correspondences between images based on descriptor similarity and geometric consistency. These correspondences provide critical geometric constraints for tasks such as 3D reconstruction [1,2], visual localization [3,4], and Simultaneous Localization and Mapping (SLAM) [5,6]. In industrial robotic applications, reliable feature matching further supports practical operational tasks including high-precision positioning, assembly, and grasping [7,8].

Typically, the feature matching pipeline consists of three steps: keypoint detection, descriptor extraction, and feature matching. Handcrafted features, such as Oriented FAST and Rotated BRIEF (ORB) [9] and Speeded-Up Robust Features (SURF) [10], rely primarily on local intensity variations. Although these methods perform well in texture-rich environments, they often suffer from insufficient repeatable keypoints on weakly textured objects commonly encountered in industrial settings. In recent years, learning-based local features, such as SuperPoint and D2-Net [11,12], have improved keypoint detection and descriptor learning through end-to-end or joint optimization. Detector-free matching methods, represented by LoFTR [13], further employ Transformers [14] to model global contextual relationships across images, thereby partially alleviating the problems of feature sparsity and limited discriminability in weakly textured scenes. However, their high computational complexity and memory overhead make them difficult to deploy on edge devices with strict real-time and resource constraints. In contrast, lightweight convolutional neural networks like XFeat [15] show strong potential for embedded deployment due to their low resource consumption and robust performance. Nevertheless, most existing lightweight methods primarily focus on 2D appearance feature learning and do not fully exploit the underlying 3D geometric information, which may lead to unstable matching under weak texture and severe near-field perspective changes.

Furthermore, most existing feature matching models are trained on outdoor long-range scenes or general-purpose vision datasets [16,17]. Industrial robots typically operate in near-field scenarios, where scale variations, perspective distortion, specular reflections, and occlusions are more pronounced. This leads to a significant domain shift between training data and real-world deployment environments [18,19]. Moreover, the surfaces of industrial workpieces often exhibit weakly textured or homogeneous material properties, providing insufficient appearance-based supervision for keypoint extraction and descriptor learning [20,21,22]. When 2D texture information lacks discriminability, relying solely on visual descriptors can easily result in mismatches and outliers. Although introducing 3D data can help, it introduces challenges such as depth noise, high acquisition costs, and increased computational burden [23,24]. Therefore, a key challenge in indoor industrial scenes is to transfer the benefits of 3D geometric supervision into lightweight 2D feature learning, while maintaining RGB-only and efficient inference during deployment.

Based on the above analysis, this paper focuses on lightweight local feature learning for indoor industrial environments. We aim to enhance the stability and discriminability of local descriptors under weakly textured, reflective, and near-field conditions through the synergistic modeling of 3D geometric and 2D textural information [25]. Similar to geometry-aware learning methods like LiftFeat [26], we focus on the enhancement of local features via geometric cues. Our approach differs by integrating robot forward kinematics, hand–eye calibration, and RGB-D observations to obtain depth-geometric information with true scale constraints. Furthermore, we design a reliability-aware geometric enhancement mechanism based on depth validity to improve the adaptability of geometric supervision and feature fusion in industrial scenarios [24,27].

We conduct comprehensive evaluations on multiple public datasets and two self-constructed datasets, covering tasks such as homography estimation, relative pose estimation, and visual localization [16,28,29,30]. Experimental results show that the proposed method improves matching robustness under near-field perspective distortion and weakly textured conditions while preserving the advantages of lightweight deployment. The model achieves competitive accuracy on both homography and pose estimation benchmarks. In addition, embedded inference tests on the NVIDIA Jetson AGX Orin platform indicate the practical utility of the proposed method as a vision front-end for industrial edge computing. The source code is available online: https://github.com/YNOGA226/GAEFeat (accessed on 3 June 2026).

The main contributions of this paper are summarized as follows:

1. We propose a robot-kinematics-supervised geometry-aware local feature learning framework. By utilizing the high-precision forward kinematics of the robot arm to obtain relative transformations between cameras, together with depth information to construct rigorous pixel-level projection mappings, the proposed method provides physically consistent supervisory signals for network training.
2. We design an explicit geometric auxiliary branch and a two-stage geometry-aware enhancement pipeline. The former guides the shared encoder to learn local surface differential geometric structures through surface normal reconstruction and cross-view geometric consistency constraints. The latter first injects gated geometric residuals into dense descriptors and then performs token-level fusion with normal priors, while optionally using reliability cues derived from depth validity and reprojection residuals to modulate attention, thereby enhancing descriptor discriminability.
3. We construct two complementary self-built datasets, comprising both simulated and physically collected samples. These datasets facilitate smoother transfer from simulation to real-world industrial scenes. Systematic experiments across multiple datasets verify the effectiveness of the proposed model in homography estimation, relative pose estimation, and visual localization, while also demonstrating its potential for edge deployment.

## 2. Related Work

### 2.1. Local Feature Learning Methods

In the field of industrial robotics, handcrafted feature algorithms, represented by ORB [9], have long been regarded as preferred front-ends for visual SLAM systems due to their computational efficiency. However, such algorithms are inherently highly dependent on the texture richness of images. When facing smooth metallic surfaces commonly found in industrial scenarios, the number of feature points extracted by traditional algorithms often drops sharply, leading to feature matching failure.

To overcome this limitation, the academic community has gradually shifted towards data-driven deep learning paradigms. In recent years, Transformer-based architectures [14] have emerged as a frontier direction for improving matching accuracy. Starting with SuperGlue [31], this class of methods leverages graph neural networks and attention mechanisms to build strong contextual associations among feature points, significantly enhancing matching robustness in extremely difficult scenes. LoFTR [13] discards the traditional feature detection step and proposes a detector-free architecture, utilizing Transformers to establish global dependencies on coarse-grained feature maps and thereby achieving robust dense matching in texture-poor regions. ASpanFormer [32] and DKM [33] further improve matching performance under severe viewpoint transformations through adaptive attention mechanisms and dense correspondence modeling. However, the aforementioned models are usually accompanied by substantial computational overhead and memory consumption, making it difficult to meet the stringent requirements of real-time on-device inference for industrial robots.

With the surging demand for edge computing, the research focus has begun to shift towards balancing performance and efficiency, giving rise to a group of lightweight matching networks. LightGlue [34] performs adaptive pruning on the attention mechanism, substantially reducing inference time while maintaining accuracy. XFeat [15] returns to the efficiency of convolutional neural networks by designing a minimalist convolutional backbone and lightweight attention modules, specifically optimized for resource-constrained devices. LiftFeat [26] attempts to introduce geometric information into lightweight models, significantly improving descriptor discriminability by fusing surface normal features obtained from monocular depth estimation. Hierarchical graph-based lightweight matching has also been explored to strengthen both local and global message passing while reducing computational cost [35]. LIM [36] adopts a lightweight CNN-based local matching architecture with efficient feature extraction and matching modules, and further improves robustness to large viewpoint and rotation changes through a rotation-aware descriptor learning design for embedded platforms. More broadly, hybrid convolutional and Transformer backbones have also been used to preserve local detail while incorporating global context in resource-constrained settings [37], which is consistent with the broader motivation for compact front-ends that maintain both local sensitivity and global representation capacity.

When these general-purpose models are transferred to near-field industrial scenarios, factors such as reflective materials, short working distances, and weakly textured surfaces amplify the gap between training data and real deployment environments, resulting in unstable feature responses and increased mismatch rates. This indicates that although existing lightweight feature models have advantages in efficiency, they still lack effective modeling of real geometric structures in complex industrial scenarios, and their industrial adaptability remains to be further improved.

### 2.2. Auxiliary Task Design Methods

To improve the robustness of feature matching algorithms in complex scenarios, existing studies have begun to enhance the feature learning process through auxiliary tasks. Conventional feature learning [38] mainly relies on descriptor-space distance constraints or matching losses. However, when dealing with weak textures and repetitive structures, such supervisory signals are often insufficient to fully constrain the geometric consistency of learned features. To address this issue, recent methods [39,40,41] have gradually introduced semantic priors, geometric consistency constraints, or multi-task learning mechanisms to improve the stability and discriminability of feature representations.

One emerging direction is to enhance feature robustness by predicting local image attributes or introducing hierarchical constraints. Neural outlier rejection methods for self-supervised keypoint learning [39] incorporate multi-view geometric consistency and learned outlier filtering into the self-supervised training loop, thereby improving the stability of keypoint learning under weak supervision. In addition, to reduce search redundancy in weakly textured regions, the A2PM framework [40] proposes a coarse-to-fine hierarchical matching strategy from regions to points. This method uses SAM [41] to generate semantic regions and formulates semantic-aware region matching as a prerequisite auxiliary task. In this way, the search space for point-level matching is constrained to high-confidence corresponding semantic regions, thereby suppressing mismatches caused by repetitive textures.

Beyond the design of specific auxiliary branches, using large-scale pretrained vision foundation models as teachers has become a new trend. MatchAnything [42] proposes a general cross-modal image matching framework. Through pretraining on large-scale synthetic data, the model obtains strong cross-sensor structural perception capability. When training lightweight models for specific industrial scenarios, high-quality pseudo-correspondences generated by MatchAnything can be introduced as auxiliary supervisory signals. This can effectively compensate for the sparsity of labels in industrial datasets and distill the general geometric understanding of large models into smaller models.

At the level of loss function design, SiLK [43] proposes a self-supervised geometric consistency objective that does not require manual annotations and directly constrains the consistency of keypoint distributions under perspective transformations. LiftFeat [26] further introduces a 3D normal constraint, penalizing matched pairs that are spatially aligned but exhibit significant differences in surface normals, thereby ensuring feature consistency on physical surfaces.

Although methods such as A2PM and MatchAnything can provide additional priors through semantic regions or large-scale pretrained models, they usually depend on complex auxiliary models or incur high inference overhead, making direct deployment difficult in resource-constrained edge systems for industrial robots. Therefore, a more suitable strategy for industrial robotic scenarios is to use available geometric supervisory signals during training to enhance the feature representation capability of compact models, while maintaining a lightweight inference process during deployment.

### 2.3. Geometry-Aware Feature Learning

Geometry-aware feature learning usually requires additional 3D geometric information as supervision or auxiliary input. Datasets represented by MegaDepth [16] mainly rely on large-scale Internet images and obtain geometric supervision through structure-from-motion and multi-view stereo reconstruction. In recent years, monocular depth estimation models such as Depth Anything [44] have also been used to generate pseudo-depth priors. However, such geometric information often cannot stably provide real physical scale, and may suffer from depth smoothing around object boundaries and under near-field viewpoint changes. In contrast, industrial robotic systems can usually acquire depth observations with physical scale through active RGB-D sensors [45] such as Intel RealSense and Kinect, providing a more direct data source for constructing geometric supervision during training.

Nevertheless, RGB-D depth observations in real industrial environments are not always reliable. Depth maps often contain holes, noise, and boundary misalignment [23,24]. To address these issues, traditional processing methods usually employ bilateral filtering or morphology-based hole-filling algorithms for depth completion [27]. However, such general-purpose image restoration methods may be counterproductive for feature matching tasks, because excessive smoothing inevitably removes high-frequency geometric details on object surfaces. Since local feature extraction highly depends on sharp gradient variations, the blurring of geometric edges can directly lead to drift in keypoint localization accuracy [46].

To enable networks to effectively utilize depth information, early studies [47,48] mostly adopted early-fusion strategies, directly concatenating depth maps and RGB images at the input stage. However, these methods usually require the model to rely on depth input during both training and inference, resulting in additional computational overhead and deployment complexity. Meanwhile, raw depth values are highly sensitive to absolute distance variations, making them difficult to generalize across different observation distances. To improve the robustness of geometric representations, DKM [33] performs dense geometry-guided correspondence modeling, while LiftFeat [26] suggests using surface normals rather than raw depth as auxiliary input. As a derived representation of depth, surface normals can describe local planarity and curvature variations more stably. Nevertheless, in near-field industrial scenarios, depth boundary noise and RGB-D alignment errors can still affect the stability of normal estimation. In addition, relying on extra geometric input during inference introduces additional computational and sensor costs, thereby reducing the deployment flexibility of the model on edge devices.

Therefore, this paper aims to transform useful geometric cues from noisy RGB-D observations into reliable training supervision, while maintaining RGB-only inference without additional computational overhead. To this end, GAEFeat derives metric-scale geometric supervision from robot kinematics, hand–eye calibration, and RGB-D observations, and enhances matching stability in weakly textured industrial scenarios through a surface-normal auxiliary branch and a reliability-aware geometry enhancement module. Table 1 further presents a comparative illustration between this work and other related methods.

## 3. Materials and Methods

**Figure 1:**
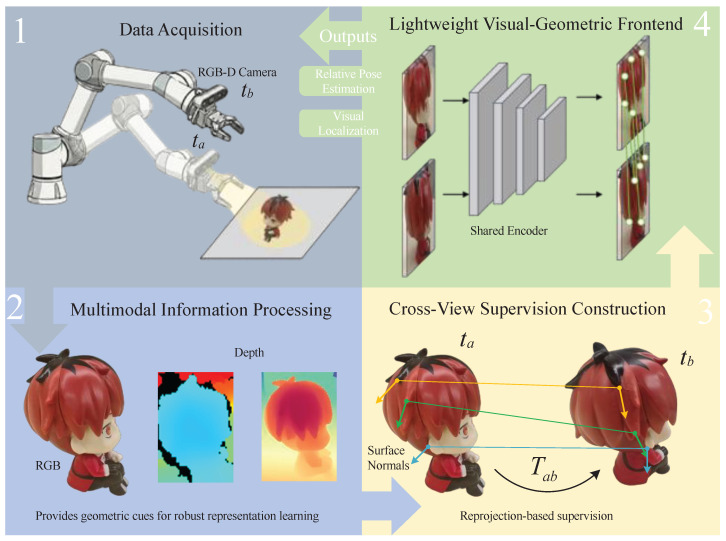
Overview of the proposed pipeline.

This section presents the overall pipeline of the proposed method, as illustrated in Figure 1. The pipeline consists of four main components: robotic data acquisition, multimodal information processing, cross-view supervision construction, and a lightweight visual–geometric front-end. First, an RGB-D camera mounted on the robotic end-effector captures RGB images, depth maps, and the corresponding robot poses of the target object from multiple viewpoints. Based on depth reprojection, we establish cross-view pixel correspondences and generate pixel-level correspondence labels together with depth reliability maps. Surface normals are then estimated from the filtered depth observations to describe local 3D surface structures and serve as geometric supervision during training. Finally, these cross-view correspondences, confidence maps, and normal cues are integrated into the feature learning process, where a geometry-aware feature fusion module improves descriptor robustness in weakly textured regions.

### 3.1. Kinematics-Guided Data and Supervision Construction

This subsection describes how the RGB-D data are acquired and converted into metric-scale geometric supervision. We first derive camera poses from robot kinematics and hand–eye calibration, then use depth-based reprojection to construct cross-view correspondence labels. We finally describe the viewpoint sampling strategy and the composition of the simulated and real datasets used for training and evaluation.

#### 3.1.1. Robotic Pose and Cross-View Reprojection

Forward kinematics allows the end-effector pose to be computed in the robot base coordinate system using joint encoder feedback. Benefiting from the high repeatability of industrial robotic arms, the pose of an RGB-D camera rigidly attached to the end-effector can be accurately represented in the robot base coordinate system for each captured frame after hand–eye calibration. This provides a reliable foundation for constructing supervision signals in close-range weakly textured scenarios. Since weakly textured objects often lack stable appearance features, supervision generation based solely on image matching or geometric reconstruction is prone to mismatches and reconstruction errors. Therefore, we employ a UR5e robotic arm (Universal Robots, Odense, Denmark) equipped with a RealSense D435i camera (Intel Corporation, Santa Clara, CA, USA) for multi-view data acquisition, and combine robot forward kinematics, hand–eye calibration, and depth reprojection to establish cross-view pixel-level correspondences.

**Figure 2:**
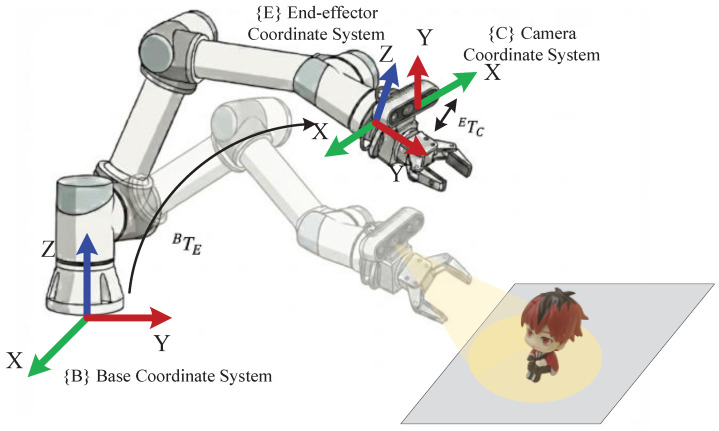
Schematic of robotic kinematics.

As shown in Figure 2, for each acquired image frame at time step *t*, the robot controller provides the pose of the end-effector frame {E} with respect to the robot base frame {B}, denoted as $T_{\mathrm{BE}}\left(t\right)$. Here, $T_{\mathrm{XY}}\in SE\left(3\right)$ denotes a homogeneous rigid transformation from coordinate frame {Y} to coordinate frame {X}. For lowercase view indices, we use the source-to-target convention; for example, $T_{ab}$ maps coordinates from source view *a* to target view *b*. Combined with the fixed camera-to-end-effector extrinsic transformation $T_{\mathrm{EC}}$, obtained from hand–eye calibration, the absolute pose of the camera frame {C} in the base frame can be further obtained as:

(1) $$T_{\mathrm{BC}}\left(t\right)=T_{\mathrm{BE}}\left(t\right)T_{\mathrm{EC}}$$

For any two observations acquired at time steps $t_{a}$ and $t_{b}$, the relative camera pose from the source view *a* to the target view *b* can then be computed from their corresponding absolute camera poses and decomposed as:

(2) $$T_{ab}={\left(T_{\mathrm{BC}}\left(t_{b}\right)\right)}^{-1}T_{\mathrm{BC}}\left(t_{a}\right)=\left[\begin{array}{cc} R_{ab} & t_{ab}\\ 0^{⊤} & 1 \end{array}\right]$$

where $R_{ab}$ and $t_{ab}$ are the relative rotation and translation from view *a* to view *b*.

For a valid depth pixel $p_{a}=\left(u_{a},v_{a}\right)$ in the source view with depth $z_{a}=D_{a}\left(p_{a}\right)$, its homogeneous coordinate is denoted as ${\tilde{p}}_{a}={\left[u_{a},v_{a},1\right]}^{⊤}$. Let $K\in R^{3\times 3}$ denote the camera intrinsic matrix. The corresponding 3D point in the source camera coordinate system is obtained by back-projection:

(3) $$P_{a}=z_{a}K^{-1}{\tilde{p}}_{a}$$

The 3D point is then transformed into the target camera coordinate system as:

(4) $$P_{b}=R_{ab}P_{a}+t_{ab}$$

The transformed point is reprojected onto the target image plane, where π(·) denotes perspective normalization:

(5) $$p_{b}=\pi \left(KP_{b}\right)$$

This process generates pixel-level cross-view supervision labels $\left(p_{a},p_{b}\right)$. During the data acquisition stage, we synchronously record the RGB image $I_{t}$, the depth map $D_{t}$, and the end-effector pose $T_{\mathrm{BE}}\left(t\right)$ for each frame. Together with the calibrated camera intrinsics K and hand–eye extrinsics $T_{\mathrm{EC}}$, these records are used to construct training-time geometric supervision, including cross-view correspondence labels derived through the reprojection process.

#### 3.1.2. Viewpoint Sampling Strategy

**Figure 3:**
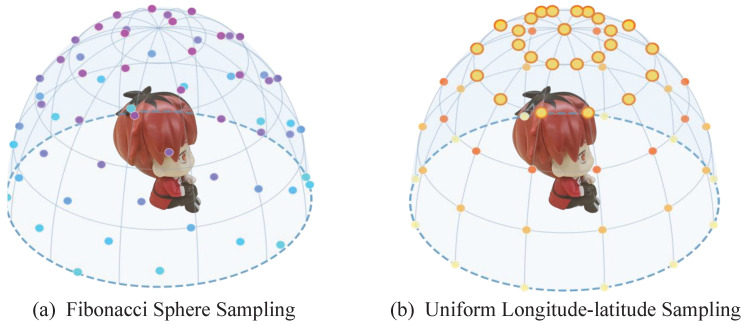
Comparison of viewpoint sampling strategies: (**a**) Fibonacci sphere sampling; (**b**) uniform longitude–latitude sampling. The colored dots denote sampled camera viewpoints and are used for visual distinction.

To generate diverse training pairs with controllable viewpoint variation, we employ a Fibonacci-sphere viewpoint sampling strategy around the object center o. Here, $o\in R^{3}$ denotes the object center in the acquisition coordinate system. Each candidate camera pose is parameterized by spherical variables (r,θ,ϕ,ψ), where *r* denotes the camera–object distance, θ the azimuth angle, ϕ the elevation angle, and ψ the in-plane roll angle around the optical axis. In accordance with the accessible workspace of the UR5e-D435i platform, we restrict these variables to

$$r\in \left[0.4,0.8\right]\;m,\;\theta \in \left[0,2\pi \right),\;\phi \in \left[\frac{\pi }{6},\frac{\pi }{2}\right],\;\psi \in \left[-\frac{\pi }{6},\frac{\pi }{6}\right],$$

which corresponds to the upper spherical-cap region illustrated in Figure 3. As shown in Figure 3, compared with uniform longitude–latitude sampling, the Fibonacci-sphere sampling strategy provides a more uniform viewpoint distribution and avoids sample clustering near the polar region. In this accessible region, we sample $N_{v}=32$ viewpoints for each object, where $N_{v}$ denotes the number of sampled camera poses. Let $\gamma =\pi (3-\sqrt{5})$ denote the golden angle. For the *i*-th viewpoint, we generate an approximately uniform viewing direction on the accessible cap by setting

$$\theta_{i}=\mathrm{mod}\left(i\gamma ,2\pi \right),\;s_{i}=s_{\min}+\frac{i+0.5}{N_{v}}\left(s_{\max}-s_{\min}\right)$$

where $i=0,\ldots ,N_{v}-1$, and $s_{i}$ denotes the vertical coordinate of the unit viewing direction, with $s_{\min}=\sin\left(\pi /6\right)$ and $s_{\max}=\sin\left(\pi /2\right)$. The corresponding elevation is recovered as $\phi_{i}=\arcsin\left(s_{i}\right)$. Given a sampled radius $r_{i}\in \left[0.4,0.8\right]\;m$, the camera center $c_{i}\in R^{3}$ is written as:

$$c_{i}=o+r_{i}\left[\begin{array}{c} \cos\phi_{i}\cos\theta_{i}\\ \cos\phi_{i}\sin\theta_{i}\\ \sin\phi_{i} \end{array}\right],\;r_{i}\in \left[0.4,0.8\right]\;m$$

The camera optical axis is oriented toward the object center through a look-at transform, while the additional roll angle $\psi_{i}$ introduces moderate in-plane rotation. This parameterization preserves approximately uniform angular coverage over the reachable upper viewing hemisphere, avoids physically implausible under-object views, and yields sampled image pairs with controllable viewpoint diversity. Candidate training pairs are further filtered by co-visibility and depth consistency before being used as supervision.

#### 3.1.3. Construction of Simulated and Real-World Datasets

To bridge the gap between general feature matching datasets and near-field weakly textured industrial scenarios, we construct two datasets in this work: a simulated dataset, Isaac-Home, and a real-world dataset, InduReal-3D. These datasets provide a metric-scale basis for training and evaluating geometry-aware feature learning methods. Specifically, Isaac-Home offers large-scale RGB-D samples with clear geometric relationships, which facilitate the learning of stable geometric matching priors. InduReal-3D further covers complex imaging conditions and physical observation uncertainties encountered in real data acquisition, improving the model’s adaptability to practical industrial scenarios.

We constructed a high-fidelity simulation environment based on NVIDIA Isaac Lab. The environment consists of ten scenes, including household scenes and factory-like workshop scenes, and contains more than 50 categories of 3D assets, such as tables, chairs, household appliances, and daily objects, forming the Isaac-Home dataset used in this work. Benefiting from the ray-tracing rendering capability of Isaac Lab, the system can realistically simulate complex illumination changes and shadow occlusions. Based on this environment, a total of 9972 RGB-D images were automatically generated by varying camera viewpoints, illumination intensity, and object textures.

**Figure 4:**
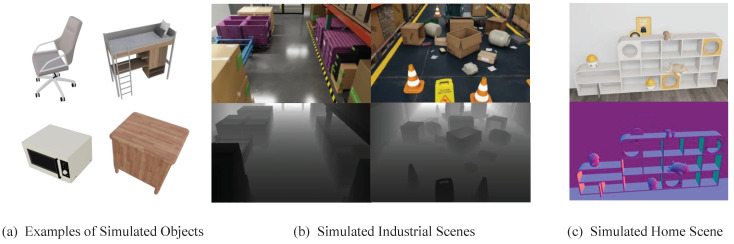
Representative examples from the simulated Isaac-Home dataset: (**a**) simulated objects; (**b**) simulated industrial scenes with RGB-D pairs; and (**c**) a simulated home scene with geometric annotations.

Figure 4 shows representative examples from the simulated dataset. The simulated subset contains reusable 3D assets, industrial-like scenes with paired RGB and depth observations, and household scenes with dense geometric annotations such as surface normals. These simulated data provide clean geometric structure and controllable pose variation, which are used to initialize the geometry-aware feature representation.

We further constructed a real-world dataset based on a physical data acquisition platform, named InduReal-3D. This dataset contains 2716 captured images from 53 physical objects, which can be grouped into several representative categories, including fruits, metallic parts, 3D-printed parts, and medicine bottles. These objects cover both everyday objects for evaluating the transfer ability from simulated scenes to real household scenarios and weakly textured or reflective industrial parts for evaluating robustness under challenging near-field conditions. The overall structure of the self-built dataset is summarized in Table 2.

**Figure 5:**
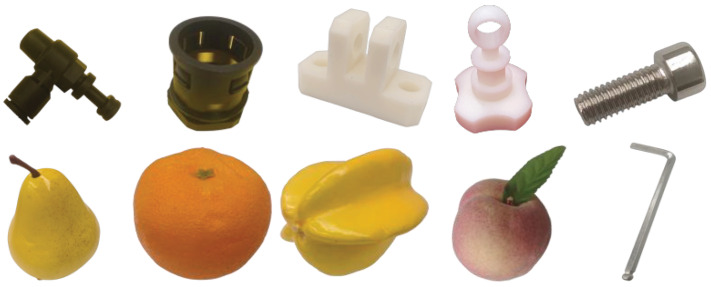
Representative objects in the real InduReal-3D dataset.

Figure 5 presents representative real objects used for data acquisition, including household objects, weakly textured parts, and reflective or metallic industrial components.

**Figure 6:**
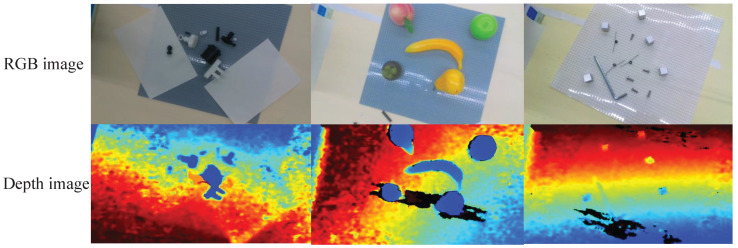
RGB-D samples from the real InduReal-3D dataset. The colors in the depth images indicate relative depth values for visualization.

Figure 6 shows representative RGB-D examples captured by the UR5e-D435i platform. Compared with the simulated data, the real depth maps contain material-dependent missing values, depth noise, and boundary artifacts, especially around reflective industrial parts and object edges. These real observations are therefore used to evaluate and fine-tune the model under practical sensing uncertainty.

### 3.2. Supervision Signal Quality Assessment

Raw depth maps often suffer from observation noise, missing values, and flying pixels at edges, making their representation of local high-frequency geometric structures vulnerable to interference. Relying solely on depth thus fails to provide stable geometric constraints. To address this, we introduce surface normals as auxiliary geometric supervision to characterize local 3D surface morphology.

To construct high-confidence geometric constraints, we apply a cascaded filtering strategy to the cross-view correspondences at both the view-pair and pixel levels. First, at the view-pair level, to eliminate candidate image pairs with minimal overlap caused by overly large baselines, given the valid-depth pixel set of source view *a*, denoted as $\Omega_{a}^{\mathrm{valid}}=\left\{p_{a}|M_{\mathrm{valid}}^{a}\left(p_{a}\right)=1\right\}$, and its reprojected subset $V_{ab}\subseteq \Omega_{a}^{\mathrm{valid}}$ that satisfies geometric visibility in target view *b*, the co-visible overlap ratio is defined as:

(6) $$r_{ab}=\frac{|V_{ab}|}{|\Omega_{a}^{\mathrm{valid}}|}$$

Only pairs where $r_{ab}$ exceeds a preset threshold $\tau_{o}$ (set to 0.20 in the experiments) are retained, thereby achieving a reasonable balance between supervision density and baseline span. The value of $\tau_{o}$ is selected on the validation split according to the Fibonacci-viewpoint sampling density and the expected co-visible region under the robot-accessible viewing range. Subsequently, at the pixel level, to precisely discard erroneous correspondences caused by occlusions, specular reflections, or sensor boundary flying pixels, a depth consistency constraint is imposed on the reprojected points within the retained set $V_{ab}$:

(7) $$\left|D_{b}\left(p_{b}\right)-{\left(P_{b}\right)}_{z}\right|<\tau_{d}$$

where ${\left(P_{b}\right)}_{z}$ is the depth component of the back-projected 3D point in the target coordinate system, and $D_{b}\left(p_{b}\right)$ is the actual depth observation at the reprojected coordinate $p_{b}$ in the target view. Reliable pixels satisfying this tolerance threshold ($\tau_{d}=0.02\;m$) are recorded using a binarized pairwise consistency mask $M_{ab}\left(p_{a}\right)\in \left\{0,1\right\}$. The value of $\tau_{d}$ is selected on the validation split as a conservative tolerance for accumulated two-view uncertainty, including depth noise, RGB-D alignment error, hand–eye calibration error, robot pose error, interpolation error, and boundary artifacts. A detailed analysis of these errors is provided below.

Beyond reprojection supervision, the filtered depth observations obtained from either simulator-rendered depth or RGB-D sensor measurements are further converted into explicit geometric supervision. Taking the source view *a* as an example, let N(a) denote the set of retained target views paired with *a*. We aggregate the pairwise consistency masks over N(a) to form a source-view support mask $M_{a}^{\mathrm{cons}}\left(p_{a}\right)$: a pixel is retained whenever it remains geometrically consistent in at least one paired target view. The filtered depth map is then obtained by applying this support mask to the original depth observation, i.e., ${\tilde{D}}_{a}\left(p_{a}\right)=D_{a}\left(p_{a}\right)\;M_{a}^{\mathrm{cons}}\left(p_{a}\right)$. In other words, ${\tilde{D}}_{a}$ is not produced by aggressive smoothing; instead, it is formed by suppressing source-view depth values that are unsupported by multi-view geometric consistency. For each valid source-view pixel $p_{a}=\left(u_{a},v_{a}\right)$, the corresponding three-dimensional point is obtained by:

(8) $$X_{a}\left(p_{a}\right)=\Pi^{-1}\;\left(p_{a},{\tilde{D}}_{a}\left(p_{a}\right)\right)={\tilde{D}}_{a}\left(p_{a}\right)\;K^{-1}{\left[u_{a},v_{a},1\right]}^{⊤}$$

where $\Pi^{-1}\left(\cdot \right)$ denotes the camera back-projection operator. Surface normals are then estimated from local finite differences on the reconstructed surface, where $\delta_{u}={\left(1,0\right)}^{⊤}$ and $\delta_{v}={\left(0,1\right)}^{⊤}$ denote unit pixel-offset vectors along the horizontal and vertical image directions:

(9) $$n_{a}\left(p_{a}\right)=\frac{\left(X_{a}\left(p_{a}+\delta_{u}\right)-X_{a}\left(p_{a}\right)\right)\times \left(X_{a}\left(p_{a}+\delta_{v}\right)-X_{a}\left(p_{a}\right)\right)}{{∥\left(X_{a}\left(p_{a}+\delta_{u}\right)-X_{a}\left(p_{a}\right)\right)\times \left(X_{a}\left(p_{a}+\delta_{v}\right)-X_{a}\left(p_{a}\right)\right)∥}_{2}}$$

To model sensor reliability for the source view, we further define a depth-reliability map:

(10) $$C_{a}\left(p_{a}\right)=M_{\mathrm{valid}}^{a}\left(p_{a}\right)\exp\;\left(-\frac{\left|D_{a}\left(p_{a}\right)-{\tilde{D}}_{a}\left(p_{a}\right)\right|}{\tau_{c}}\right)$$

where $D_{a}$ denotes the original depth observation in view *a*, $M_{\mathrm{valid}}^{a}$ denotes the valid-depth mask of view *a*, and $\tau_{c}$ is the reliability decay parameter, which is fixed to 0.008m. The value of $\tau_{c}$ is selected on the validation split as the exponential decay scale for depth reliability. With $\tau_{c}=0.008\;m$, mild depth fluctuations remain partially weighted, while larger residuals caused by reflection, boundary misalignment, or multi-view inconsistency are strongly downweighted before reaching the hard rejection threshold $\tau_{d}$. This formulation assigns high confidence to pixels whose original depth remains stable after multi-view consistency filtering, while downweighting pixels affected by holes, boundary flying pixels, reflective noise, or RGB-D misalignment. All three thresholds are fixed for all reported experiments without tuning on the test set. For substantially different RGB-D sensors, working distances, depth-noise levels, or object scales, these values should be revalidated according to the actual depth-residual distribution, object image coverage, and viewpoint sampling density.

**Figure 7:**
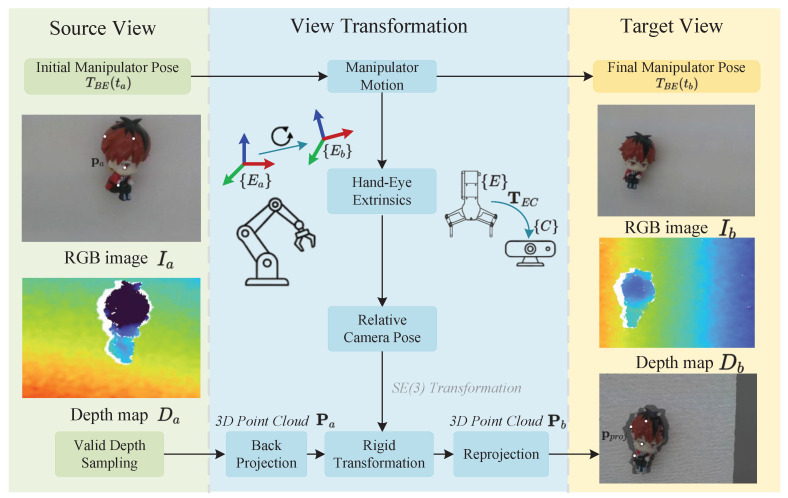
Kinematics-guided cross-view correspondence generation pipeline. The colors in the depth maps indicate relative depth values for visualization.

Figure 7 summarizes the above projection-based generation process. Given a source RGB-D observation, valid depth pixels are back-projected into 3D space, transformed by the relative camera pose derived from robot kinematics and hand–eye calibration, and reprojected onto the target view to establish candidate cross-view correspondences. The retained geometrically consistent regions are then used to derive correspondence labels, surface-normal labels, and depth reliability maps.

Based on the aforementioned generation process of cross-view reprojection correspondences, surface-normal labels, and depth reliability maps, we further analyze the quality of the supervision signals. Reliable supervision signals should satisfy true geometric relationships as much as possible: cross-view reprojection correspondences should reflect the true projection relationship of the same 3D surface point across different views, and surface-normal labels should characterize the true orientation changes of the local surface, rather than being dominated by anomalous factors such as depth noise, boundary flying pixels, specular interference, or RGB-D alignment errors.

For the UR5e-D435i data acquisition system utilized in this work, the quality of the supervision signals is primarily affected by three types of errors. The first type is the relative camera pose deviation caused by robot kinematics and hand–eye calibration, which directly affects the reprojection locations of 3D points from the source view to the target view. The second type is the pixel-level geometric deviation caused by RGB-D alignment errors and depth measurement noise; this type of error leads to inconsistencies between RGB boundaries and depth boundaries, further affecting the depth consistency judgment at reprojected points. The third type is the local depth degradation caused by object boundaries, specular reflection regions, and depth holes. Such errors disrupt local depth continuity and may be further amplified during the surface normal estimation process. The roles of the recorded data and derived supervisory signals during training and inference are summarized in Table 3.

In our system, these error sources are bounded within a relatively small but non-negligible range under the adopted near-field acquisition setup. Within the operating range of approximately 0.4–0.8m, the UR5e repeatability is 0.05mm; the hand–eye calibration error is controlled within approximately 0.5mm in translation and 0.10° in rotation; the RGB-depth alignment deviation is about 0.5 pixels near the image center and may increase to roughly 2 pixels near the image boundary; and the D435i depth error is typically around 2mm on diffuse surfaces but may rise to about 5mm in reflective regions, missing-depth regions, and object boundaries. In addition, image acquisition is triggered only after the manipulator reaches a stationary state, making synchronization errors negligible in practice. These residual errors motivate the subsequent co-visibility and depth-consistency filtering strategy, rather than being ignored in the supervision design.

**Figure 8:**
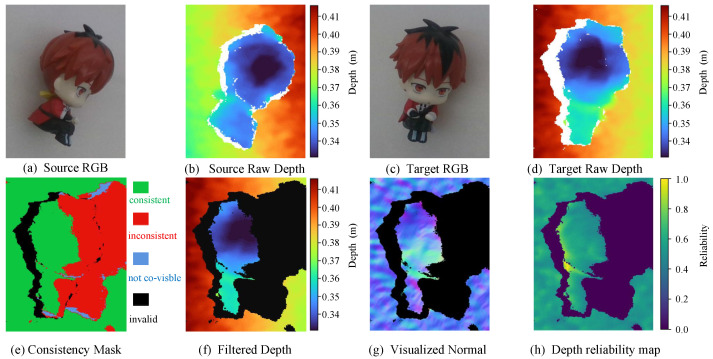
Visualization of supervision signal quality. (**a**) Source RGB image; (**b**) source raw depth; (**c**) target RGB image; (**d**) target raw depth; (**e**) consistency mask; (**f**) filtered depth; (**g**) visualized surface normal; and (**h**) depth reliability map.

As intuitively reflected in Figure 8, the raw depth maps in Figure 8b,d can delineate the rough depth distribution of the target but exhibit obvious depth discontinuities at object contours, background boundaries, and locally missing regions. These regions typically correspond to RGB-D alignment errors, boundary flying pixels, specular interference, or depth measurement failures. If directly involved in supervision generation, they are likely to compromise the reliability of cross-view correspondences and surface normal estimation.

After multi-view consistency filtering, the consistency mask in Figure 8e effectively distinguishes geometrically stable regions from unreliable ones. The resulting supported valid-depth region, shown in Figure 8f, preserves the primary depth structure of the target surface while mitigating the influence of boundaries and anomalous areas. The surface-normal labels generated from these filtered depth observations, shown in Figure 8g, exhibit a relatively continuous orientation distribution within reliable regions and better reflect local surface variations. Meanwhile, the depth reliability map in Figure 8h explicitly downweights low-confidence regions, allowing network training to rely more on areas with high geometric consistency.

These visualization results demonstrate that our supervision signals are not directly derived from raw depth maps, but are deliberately constructed through consistency filtering and reliability modeling. The reprojection correspondences, surface-normal labels, and reliability maps together provide a more stable form of geometric supervision, effectively reducing the interference of depth noise during network training.

### 3.3. Network Architecture

**Figure 9:**
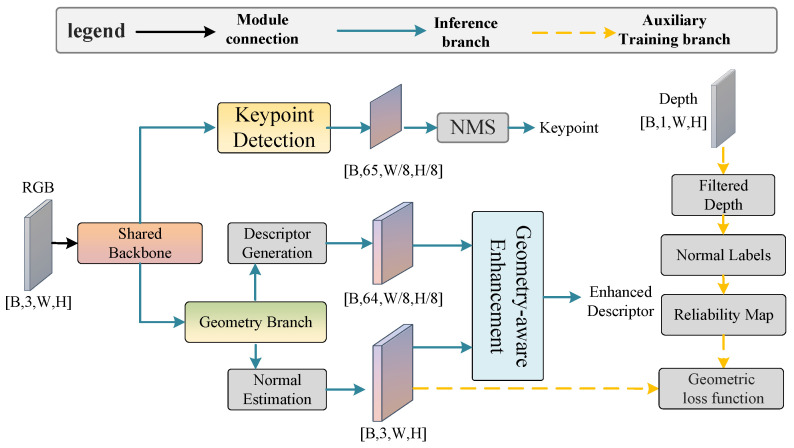
Refined overall architecture of GAEFeat.

Considering the limited computational resources of edge devices, we propose the GAEFeat network architecture shown in Figure 9. The architecture explicitly separates the pure RGB inference path used during deployment from the auxiliary geometric supervision signals introduced only during training. Its backbone adopts a fully convolutional design, and the overall framework consists of a shared feature extraction backbone, the Keypoint Detection module, and a geometry-aware descriptor-learning pipeline composed of the Geometry Branch, Descriptor Generation, Normal Estimation, and geometry-aware enhancement modules.

To make the data flow explicit, we describe the main modules according to their inputs, outputs, and supervision signals. The shared backbone takes the RGB image as input and outputs the shared feature representation $F_{\mathrm{sh}}$. The Keypoint Detection module takes $F_{\mathrm{sh}}$ as input, predicts the keypoint heatmap, and is supervised by the keypoint loss $L_{\mathrm{kpt}}$. The descriptor-learning pathway then uses $F_{\mathrm{sh}}$ to construct both appearance descriptors and geometry-aware cues. Specifically, the Descriptor Generation module maps $F_{\mathrm{sh}}$ to the basic dense descriptor map D, which serves as the descriptor substrate before geometric refinement, while the Geometry Branch derives the geometry-sensitive feature G and the gated refined descriptor $D_{\mathrm{ref}}$ from this descriptor representation. The Normal Estimation head predicts the normal prior $\hat{N}$ from G and is supervised by the single-view normal loss $L_{\mathrm{normal}}$ and the cross-view consistency loss $L_{\mathrm{cv}}$. Finally, the geometry-aware enhancement module takes $D_{\mathrm{ref}}$ and $\hat{N}$ as inputs, performs reliability-aware visual–geometric fusion, and outputs the enhanced dense descriptor map from which the final sparse descriptors are sampled and optimized by $L_{\mathrm{desc}}$.

**Figure 10:**
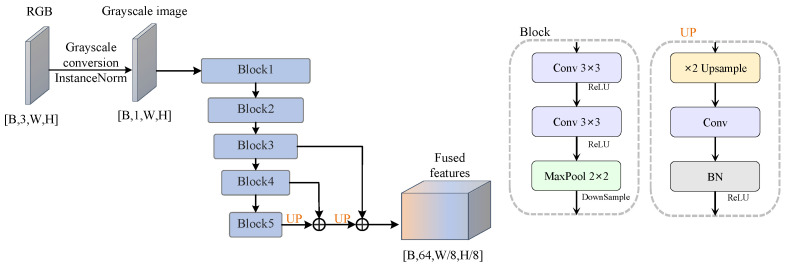
Detailed structure of the shared backbone.

GAEFeat takes a single RGB image as input. Following the implementation illustrated in Figure 10, the backbone first converts the RGB image into a grayscale image and normalizes it with InstanceNorm2d to suppress near-field illumination fluctuations and appearance bias. The encoder is then composed of five consecutive convolutional blocks (Block1–Block5), where each block follows a Conv-ReLU-Conv-ReLU-MaxPool design to jointly perform feature extraction, channel expansion, and spatial downsampling. The basic upsampling unit, denoted as UP, adopts an Upsample-Conv-BN-ReLU structure. To mitigate the loss of spatial details caused by downsampling, the model adopts a feature pyramid fusion structure. Specifically, the high-level semantic features from Block5 are upsampled and fused along the channel dimension with the intermediate features from Block3 and Block4, thereby producing a shared feature representation.

The shared representation is propagated along two downstream paths. One path is fed directly into the Keypoint Detection module, which adopts a lightweight grid-classification strategy to balance real-time performance and localization accuracy. During inference, full-resolution keypoint heatmaps are recovered through channel-wise Softmax and sub-pixel rearrangement, thereby reducing computational overhead and avoiding checkerboard artifacts. The other path forms the dense descriptor-learning pathway. In near-field industrial scenes, where weak-texture surfaces and homogeneous backgrounds are common, descriptors relying solely on RGB information often fail to capture sufficiently discriminative matching structures. To establish a stable descriptor substrate, the shared feature $F_{\mathrm{sh}}$ is first mapped by the Descriptor Generation module to a basic dense descriptor map $D=H_{d}\left(F_{\mathrm{sh}}\right)$.

**Figure 11:**
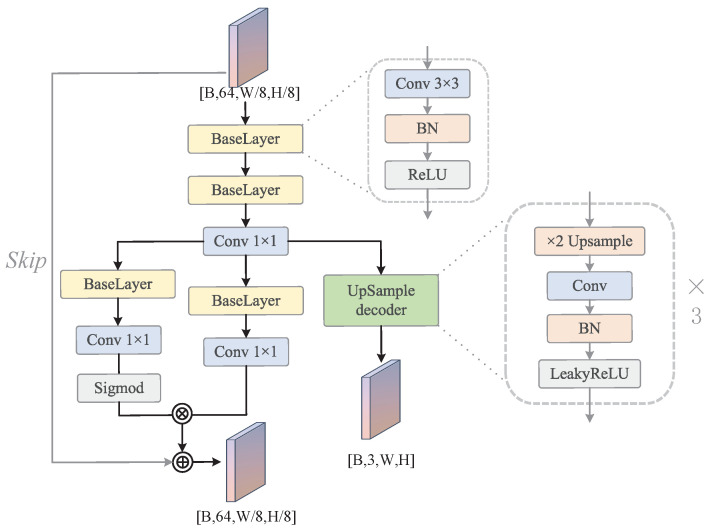
Detailed structure of the Geometry Branch.

As further detailed in Figure 11, the basic descriptor D is not directly used as the final matching representation. Instead, it is passed to the Geometry Branch, where a geometry feature head implemented by two stacked BaseLayers and a 1×1 projection reorganizes the local descriptor into a geometry-sensitive feature space. The first-stage refinement can be summarized as

(11) $$G=H_{g}\left(D\right)$$

(12) $$D_{\mathrm{ref}}=D+\sigma \;\left(H_{m}\left(G\right)\right)⊙H_{r}\left(G\right)$$

(13) $$\hat{N}=H_{n}\left(G\right)$$

where $H_{d}\left(\cdot \right)$ denotes the descriptor generator, $H_{g}\left(\cdot \right)$ the geometry feature head, $H_{m}\left(\cdot \right)$ and $H_{r}\left(\cdot \right)$ the gate and residual projections, and $H_{n}\left(\cdot \right)$ the auxiliary normal-prediction head. This first-stage residual refinement preserves essential appearance details through the skip connection while selectively enhancing responses supported by reliable local geometry.

**Figure 12:**
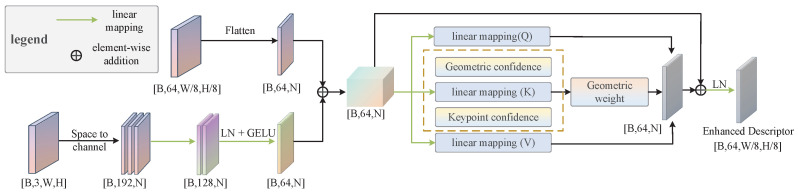
Detailed structure of the geometry-aware enhancement module.

During training, the depth-derived normal labels from Section 3.1 supervise the auxiliary normal head, while the corresponding depth-reliability map provides cues for judging which geometric responses should be trusted. The first-stage outputs used by the second-stage enhancement are therefore the refined dense descriptor map $D_{\mathrm{ref}}$ and the predicted normal map $\hat{N}$. Following Figure 12, the explicit geometric prior used by the second-stage enhancement module is the normal tensor itself, written as $I_{\mathrm{geo}}=\hat{N}$. Geometry confidence is derived from intermediate gating responses, residual statistics, and reliability cues inside the first-stage Geometry Branch, and used as an auxiliary weight when modulating feature fusion.

To further alleviate the degradation of feature discriminability caused by weak-texture objects and sensor noise in near-field industrial environments, we design a geometry-aware enhancement module that performs deep fusion of texture features and geometric priors through an attention mechanism. Its refined structure is illustrated in Figure 12, which highlights the normal-driven token fusion together with the optional confidence-guided weighting applied before descriptor enhancement.

In the geometry-aware enhancement module, the refined dense descriptor map is flattened into a sequence representation, written as $F_{\mathrm{tex}}=\mathrm{Flatten}\left(D_{\mathrm{ref}}\right)$. In the geometric-prior branch, the predicted normal map $I_{\mathrm{geo}}=\hat{N}$ is rearranged by a space-to-channel operator to collect local surface-orientation cues and is then projected into the same feature space as the texture branch. With the local window size fixed by the implementation, this produces a token-level geometric encoding $H_{\mathrm{geo}}=S2C\left(I_{\mathrm{geo}}\right)\in R^{N\times 192}$, which preserves neighborhood-level surface orientation context rather than isolated per-pixel normals. The core normal-driven fusion in the second stage is written as

(14) $$F_{\mathrm{geo}}=\mathrm{GELU}\;\left(\mathrm{LN}\;\left(W_{g}\;H_{\mathrm{geo}}\right)\right)$$

(15) $$A=\sigma \;\left(W_{c}\;H_{\mathrm{geo}}\right)$$

(16) $$F_{\mathrm{fuse}}=F_{\mathrm{tex}}+A⊙F_{\mathrm{geo}}$$

where $W_{g}$ and $W_{c}$ are learnable projection matrices, LN(·) denotes layer normalization, σ(·) is the sigmoid function, ⊙ denotes element-wise multiplication, A is the learned fusion gate, and *N* is the number of tokens after flattening. The dashed boxes in Figure 12 correspond to optional geometry-confidence and keypoint-confidence weights: the former is derived from the first-stage Geometry Branch, whereas the latter is provided by the keypoint-response stream. When enabled, these auxiliary weights further reweight the gate before attention aggregation; otherwise, the default forward path is driven by the predicted normal prior alone. Unless otherwise specified, these auxiliary weights remain enabled in all subsequent experiments.

The fused tokens are then processed by the lightweight attention-based enhancement stage and reshaped back to an enhanced dense descriptor map. During inference, keypoints are obtained from the heatmap predicted by the Keypoint Detection branch through non-maximum suppression, while local descriptors are sampled from the enhanced dense descriptor map at the retained keypoint locations. In this way, geometric enhancement is performed on the dense descriptor field before sparse feature extraction, preserving the efficient RGB-only inference mode of the overall front-end.

### 3.4. Loss Functions

In the design of the keypoint detection loss, considering the real-time constraints of edge-side deployment in industrial robotics, we do not adopt a full-resolution heatmap regression scheme with high computational overhead. Instead, the localization task is reformulated as an efficient grid-classification problem. Specifically, the input image is partitioned into local grids, and the network predicts the probability distribution over 64 pixel locations within each grid as candidate keypoints. The 65th channel is used for background suppression. Benefiting from the high-precision pose ground truth provided by robot-arm kinematics, we can automatically assign the true location label to each grid through pixel-level reprojection consistency verification. This process is supervised by a cross-entropy loss:

(17) $$L_{\mathrm{kpt}}=-\frac{64}{WH}\sum_{i,j}\sum_{k=1}^{65}y_{i,j,k}\log\;\left(P_{i,j,k}\right)$$

where *W* and *H* denote the input image width and height, (i,j) indexes a local grid cell, *k* indexes one of the 65 classification channels, $P_{i,j,k}$ is the predicted probability, and $y_{i,j,k}$ is the corresponding one-hot label generated from the physical pose ground truth. For the descriptor loss, the objective is to preserve consistency in the feature representation space. In this work, point pairs that are close in feature space but do not correspond in physical space are explicitly constrained, thereby improving descriptor discriminability:

(18) $$L_{\mathrm{desc}}=\sum \max\;\left(0,\;d{\left(f_{A},f_{B}\right)}^{2}-d{\left(f_{A},f_{\mathrm{neg}}\right)}^{2}+m\right)$$

where d(·,·) denotes the Euclidean distance in descriptor space, $f_{A}$ and $f_{B}$ are descriptors of a matched point pair, $f_{\mathrm{neg}}$ is a negative descriptor, and *m* is the triplet margin. Supervised learning under physical constraints enables GAEFeat to embed the spatial transformation patterns perceived by the robot arm into the learned feature representation, thereby improving matching robustness.

Relying only on local keypoint supervision and descriptor discrimination may still cause the network to overfit unstable appearance cues in weak-texture and specular regions. We therefore introduce a geometric auxiliary loss composed of single-view normal supervision and cross-view geometric consistency:

(19) $$L_{\mathrm{geo}}=\lambda_{\mathrm{normal}}L_{\mathrm{normal}}+\lambda_{\mathrm{cv}}L_{\mathrm{cv}}.$$

Let ${\hat{n}}_{a}\left(p_{a}\right)$ denote the unit normal predicted by the auxiliary Geometry Branch at source pixel $p_{a}$, $n_{a}^{\mathrm{gt}}\left(p_{a}\right)$ the unit surface-normal label derived from depth as defined in Section 3.1, and $M_{a}\left(p_{a}\right)$ the valid supervision mask. The single-view normal supervision loss is defined as:

(20) $$L_{\mathrm{normal}}=\frac{\sum_{p_{a}}M_{a}\left(p_{a}\right)\left(1-\langle {\hat{n}}_{a}\left(p_{a}\right),n_{a}^{\mathrm{gt}}\left(p_{a}\right)\rangle \right)}{\sum_{p_{a}}M_{a}\left(p_{a}\right)}$$

This term encourages the shared encoder to capture stable local surface orientation even when texture information is insufficient.

To enforce cross-view geometric consistency, the predicted normal in the source view is rotated into the target camera coordinate system according to the reprojection relation defined in Section 3.1. For a source–target pair a→b, the cross-view consistency loss is written as:

(21) $$L_{\mathrm{cv}}^{a\to b}=\frac{\sum_{p_{a}}M_{ab}\left(p_{a}\right)\left(1-\langle {\hat{n}}_{a\to b}\left(p_{a}\right),n_{b}^{\mathrm{gt}}\left(p_{a\to b}\right)\rangle \right)}{\sum_{p_{a}}M_{ab}\left(p_{a}\right)}$$

where ${\hat{n}}_{a\to b}\left(p_{a}\right)=R_{ab}{\hat{n}}_{a}\left(p_{a}\right)$ denotes the predicted normal from the source view after rotation into the target camera frame, $p_{a\to b}$ denotes the corresponding target pixel obtained by reprojection, and $M_{ab}\left(p_{a}\right)$ is the joint valid mask that is equal to 1 only in regions that are co-visible, projectable, and geometrically reliable. The symmetric cross-view loss is then defined as

(22) $$L_{\mathrm{cv}}=\frac{1}{2}\left(L_{\mathrm{cv}}^{a\to b}+L_{\mathrm{cv}}^{b\to a}\right)$$

Finally, the network is trained end-to-end using the following multi-task joint loss:

(23) $$L_{\mathrm{total}}=\lambda_{\mathrm{kpt}}L_{\mathrm{kpt}}+\lambda_{\mathrm{desc}}L_{\mathrm{desc}}+\lambda_{\mathrm{geo}}L_{\mathrm{geo}}$$

## 4. Experiments

We evaluate GAEFeat on three tasks: relative pose estimation, homography estimation, and visual localization. The quantitative comparisons, ablation studies, and runtime analysis are reported below.

### 4.1. Experimental Setup

We implement the proposed method in PyTorch 1.13.1. The training data include MegaDepth [16], a COCO_20*k*_ subset of the COCO2017 training set [17], and the training splits of the hybrid RGB-D dataset, including the simulated Isaac-Home subset and the real InduReal-3D subset. Input images are resized to 800 × 600 pixels. Following LiftFeat, the generic pretraining stage is optimized with Adam using an initial learning rate of $3\times 10^{-4}$ and a StepLR scheduler with decay factor 0.5 every 10,000 iterations. During training, 1024 matched point pairs are sampled to refine the feature aggregation module.

Considering the computational constraints of robotic systems, we compare against lightweight baselines including ORB [9], SuperPoint [11], ALIKE [49], SiLK [43], and XFeat [15]. For SiLK and ALIKE, the smallest available backbones are used so that the comparison focuses on efficiency-oriented models. For all baselines, the top 4096 detected keypoints are retained, and mutual nearest-neighbor search is used for matching.

The public benchmarks used in the experiments cover different challenging conditions. MegaDepth-1500 mainly evaluates outdoor wide-baseline matching with large viewpoint changes, scale variations, illumination changes, occlusions, and complex scene depth distributions, whereas ScanNet focuses on indoor RGB-D scenes with weakly textured surfaces, clutter, repetitive structures, motion blur, and RGB-D sensing noise. For Aachen Day-Night v1.1, we follow the standard visual-localization protocol [30] and report recall at 0.25 m/2°, 0.5 m/5°, and 5 m/10°, corresponding to strict, medium, and coarse localization thresholds that are widely used in prior work.

The experiments aim to evaluate the robustness and real-time performance of GAEFeat in industrial near-field environments. Training is conducted on a platform equipped with 16 vCPUs, dual NVIDIA RTX 4090 GPUs, Ubuntu 22.04, Python 3.8, PyTorch 1.13.1, and CUDA 11.8. To validate practical deployability, inference speed and real-time performance are further evaluated on the NVIDIA Jetson AGX Orin embedded platform.

To reduce the risk of data leakage, we adopt category-level or scene-level splitting rules when partitioning the simulated and real-world RGB-D datasets. For the real-world InduReal-3D dataset, the 3D-printed-part category is held out for testing, while the remaining object categories, including fruits, metallic parts, and medicine bottles, are used for training and validation. This category-level split ensures that the model is evaluated on an unseen object category rather than on adjacent viewpoints or different instances from categories observed during training. For the simulated Isaac-Home dataset, the split is performed at the scene level. Among the ten simulated scenes, one factory scene is reserved as the test scene, while the remaining household and factory scenes are used for training and validation. All image pairs are generated only within the same split, and no cross-split image pair is used for training or evaluation. The validation splits are used only for model selection and hyperparameter checking, while the test splits are kept unseen during parameter optimization. The resulting split statistics and acquisition conditions are summarized in Table 4.

This work adopts a progressive transfer-learning strategy that largely follows LiftFeat in the generic pretraining stage and then extends it to industrial RGB-D supervision. Similar to LiftFeat, the first stage trains the encoder on MegaDepth and the COCO_20*k*_ subset to learn transferable local matching priors from large-scale natural imagery. In practice, we follow the same mixed-data recipe with $1\times 10^{5}$ optimization steps, a MegaDepth batch size of 16, and a COCO batch size of 8. The role of this stage is to establish stable appearance-driven keypoint detection and descriptor extraction before any task-specific geometry is introduced. Starting from this initialization, we introduce a high-fidelity simulation environment built on NVIDIA Isaac Lab for industrial scenes. By leveraging the noise-free depth maps and pose ground truth provided by the simulator, we derive surface-normal labels and depth-reliability masks for privileged supervision and reliability-gated fusion.

This geometry-aware stage is trained for 50 epochs using the training split of the simulated Isaac-Home dataset together with pixel-level pose ground truth. Its role is to inject explicit near-field geometric priors into the shared encoder under clean and physically controllable supervision. Finally, the model is fine-tuned for 30 epochs only on the training split of the self-built real InduReal-3D dataset with a batch size of 8, using millimeter-level pose constraints from robotic kinematics. During fine-tuning, composite data augmentation, including specular-reflection simulation and speckle-noise injection, is used to improve robustness in reflective industrial environments. This final stage mainly reduces the remaining domain gap caused by real sensor noise, reflective materials, and calibration imperfections. The InduReal-3D validation split is used for model selection and hyperparameter checking, and the InduReal-3D test split remains unseen until final evaluation. After all scheduled training stages described above are completed, the model parameters are fixed for evaluation. No additional fine-tuning on evaluation or test splits and no test-time adaptation are performed. The geometry-specific hyperparameters are fixed throughout all reported experiments: the depth-consistency threshold is set to $\tau_{d}=0.02\;m$, the reliability decay factor is set to $\tau_{c}=0.008\;m$, the task-loss weights are $\lambda_{\mathrm{kpt}}=1$, $\lambda_{\mathrm{desc}}=1$, and $\lambda_{\mathrm{geo}}=0.8$, and the geometric-loss weights are $\lambda_{\mathrm{normal}}=1$ and $\lambda_{\mathrm{cv}}=0.5$.

### 4.2. Benchmark Results

This section reports quantitative results on relative pose estimation, homography estimation, and visual localization, and then provides ablation, efficiency, and qualitative analyses to further examine the effectiveness of the proposed geometry-aware design in complex environments.

In our experimental setup, we follow the standard protocol of XFeat by resizing MegaDepth images to a maximum resolution of 1200 pixels, while ScanNet is evaluated at VGA resolution. Following mainstream evaluation protocols, we use the MAGSAC++ robust estimator [50] to estimate the fundamental matrix and report the area under the recall curve (AUC) of pose estimation errors under different thresholds. As shown in Table 5, GAEFeat achieves performance comparable to LiftFeat on MegaDepth-1500 and obtains the best results on ScanNet under all three thresholds. These results indicate that the proposed geometry-aware design is particularly beneficial in indoor RGB-D scenarios, where geometric structure and depth-related cues are more relevant.

We further evaluate homography estimation on the widely used HPatches dataset [29], which consists of planar image sequences with diverse illumination and viewpoint changes. Each sequence contains five image pairs and their ground-truth homography matrices. The evaluation metric is Mean Homography Accuracy (MHA), i.e., the proportion of image pairs whose reprojection error falls below a given pixel threshold of {3, 5, 7}. Table 6 reports the results on HPatches [29] under illumination and viewpoint changes. GAEFeat achieves competitive performance under illumination changes and shows a slight advantage in the viewpoint split. This result suggests that the explicit geometric auxiliary branch is especially helpful when planar matching must remain stable under larger viewpoint variation.

We evaluate the visual localization performance of GAEFeat on the challenging Aachen Day-Night v1.1 benchmark following the standard evaluation protocol [30]. The dataset contains 6697 daytime database images and 1015 query images, including 191 nighttime queries, making it suitable for assessing robustness under severe illumination changes. Localization accuracy is reported as the percentage of query images whose estimated camera poses fall within the standard translation/rotation error thresholds described in Section 4.1. In our experiments, the maximum image size is set to 1024 pixels, and up to 4096 keypoints are extracted for each image.

As shown in Table 7, GAEFeat performs competitively against lightweight baselines in both day and night scenes. It achieves the best recall in daytime localization and remains comparable to LiftFeat at night, including a slight advantage under the strictest nighttime threshold. These results indicate that GAEFeat does not sacrifice localization robustness under illumination changes, although its nighttime advantage over LiftFeat remains limited.

As shown in Table 8, GAEFeat achieves the best relative pose estimation results on both the held-out Isaac-Home simulated test split and the real-world InduReal-3D test split. On Isaac-Home, GAEFeat reaches 51.7, 71.2, and 84.6 at 5°, 10°, and 20°, respectively, outperforming the strongest lightweight baseline, LiftFeat, by +3.5, +4.3, and +5.4 AUC. On InduReal-3D, GAEFeat obtains 19.7, 37.2, and 51.7 under the same thresholds, improving over LiftFeat by +1.8, +2.6, and +3.6 AUC. These results suggest that geometry-aware training improves matching robustness in simulated household and workshop-like scenes and transfers effectively to real near-field industrial scenes with sensor noise, reflective surfaces, and weak texture.

### 4.3. Ablation and Efficiency Analysis

We conduct a stepwise ablation study on the challenging nighttime visual-localization test set to evaluate the contribution of each geometric component. As shown in Table 9, introducing the geometric auxiliary branch improves the recall under the strictest threshold from 78.8% to 79.9%, indicating that geometric supervision enhances the robustness of the learned local representation. Adding the geometry-aware enhancement module without the reliability gate further increases the recall to 80.9%, 88.7%, and 98.6%, showing that normal-driven feature fusion brings additional gains over auxiliary supervision alone. The full GAEFeat setting further enables reliability-aware confidence weighting and achieves 82.3%, 89.6%, and 99.0%, respectively. Compared with the variant without the reliability gate, the full model improves the recall by +1.4, +0.9, and +0.4 points under the three localization thresholds, indicating that reliability-aware fusion provides consistent additional gains and is especially useful under the strictest nighttime localization criterion, where low-light, reflective, and noisy regions are more likely to introduce misleading geometric cues.

We benchmark the feature-extraction cost on an embedded platform equipped with an 8-core Arm Cortex-A78AE CPU and an NVIDIA Jetson AGX Orin GPU. For computational profiling, all models are evaluated with VGA-resolution input images of 640×480 pixels. The FLOPs reported in Table 10 are measured using a PyTorch-based profiler, following the standard MAC (multiply–accumulate) convention. The latency denotes single-image network inference time for the feature-extraction forward pass, excluding mutual nearest-neighbor matching, geometric verification, and pose estimation. Although the reliability-aware geometry enhancement makes GAEFeat slightly heavier than LiftFeat, the additional cost is limited: the FLOPs increase from 4.96G to 5.37G, and the GPU latency increases from 3.7 ms to 3.9 ms. In return, this design improves the robustness of feature matching under weak-texture, reflective, and geometrically degraded conditions, which are common in the target near-field industrial scenarios.

We conduct an ablation study on the InduReal-3D Test test to evaluate the effectiveness of the proposed geometric auxiliary loss in industrial near-field matching. As shown in Table 11, the values in parentheses denote absolute gains over the baseline using only $L_{\mathrm{kpt}}$ and $L_{\mathrm{desc}}$. Introducing the single-view normal loss $L_{\mathrm{normal}}$ produces the major gain, improving AUC@20° from 44.5 to 50.9 and confirming that depth-derived surface-normal labels provide strong local structural guidance in weak-texture scenes. Adding the cross-view consistency loss $L_{\mathrm{cv}}$ further raises the score to 51.7, indicating that enforcing geometric agreement across viewpoints yields an additional, though smaller, gain in correspondence stability under reflective and ambiguous industrial conditions.

We conduct a simulated-scene scaling ablation by varying the number of Isaac-Home scenes used during the geometry-aware simulation training stage to further examine the scalability of the automated simulation-labeling pipeline. Different from random image-level subsampling, each subset is constructed at the scene level, so that the experiment reflects both the amount of simulated data and the diversity of simulated environments while avoiding leakage from adjacent viewpoints in the same scene. The held-out factory scene in Isaac-Home is never used for training. In the main scaling rows, the real InduReal-3D fine-tuning split is kept fixed at 2036 images, so that the effect of increasing simulated scene diversity can be isolated. We additionally include two endpoint controls without real fine-tuning or without simulated training to clarify the complementary roles of synthetic geometric supervision and real-domain adaptation.

Table 12 evaluates the contribution of simulated and real training data on the held-out InduReal-3D test split. Both Isaac-Home training and real-domain fine-tuning improve the generic model, and their combination achieves the best performance, with AUC values of 19.7, 37.2, and 51.7 at the 5°, 10°, and 20° thresholds. As more Isaac-Home scenes are added, performance generally improves but gradually saturates at larger simulation scales. These results suggest that simulated geometric supervision and real-domain adaptation are complementary, supporting the scalability of the simulation-labeling pipeline while highlighting the need for real-domain fine-tuning to close the remaining domain gap.

### 4.4. Qualitative Results

**Figure 13:**
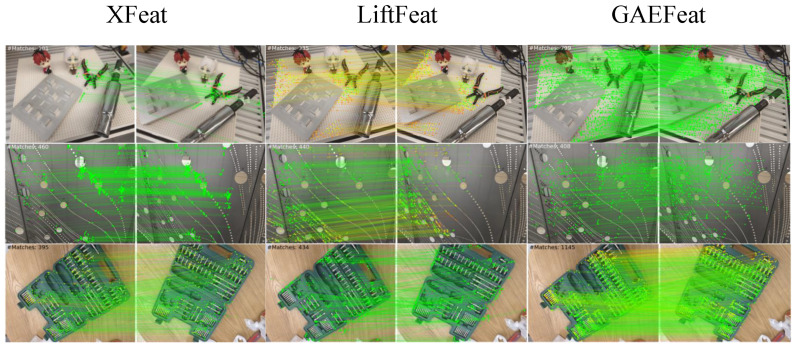
Qualitative comparison in representative scenarios. Colored lines and points visualize feature correspondences.

Figure 13 presents qualitative comparisons in representative scenarios. In the real-household transfer case shown in the first row, GAEFeat benefits from simulation pretraining on Isaac-Home and produces denser geometrically consistent correspondences on complex real household objects such as cluttered figurines and pliers than XFeat. This suggests that the learned representation is not overly tied to specific appearance patterns and retains transferable geometric structure. The second and third rows further show that this advantage remains visible on reflective indoor surfaces and weak-texture industrial parts, where GAEFeat preserves denser and more spatially coherent correspondences than the compared lightweight baselines.

**Figure 14:**
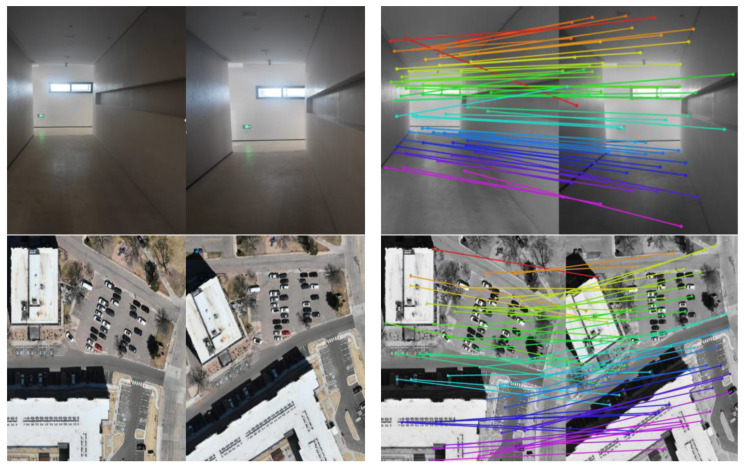
Additional general-scene matching examples under viewpoint change. Colored lines and points visualize feature correspondences.

As supplementary general-scene examples, Figure 14 presents two cross-view matching cases: an indoor corridor with moderate viewpoint change and an aerial-view urban scene. Although these examples are less industrial than the reflective near-field scenarios in Figure 13, they still show stable long-range correspondences along weak-texture structural regions. This observation suggests that the proposed geometry-aware training improves robustness in challenging industrial conditions without sacrificing matching behavior in more general indoor scenes.

**Figure 15:**
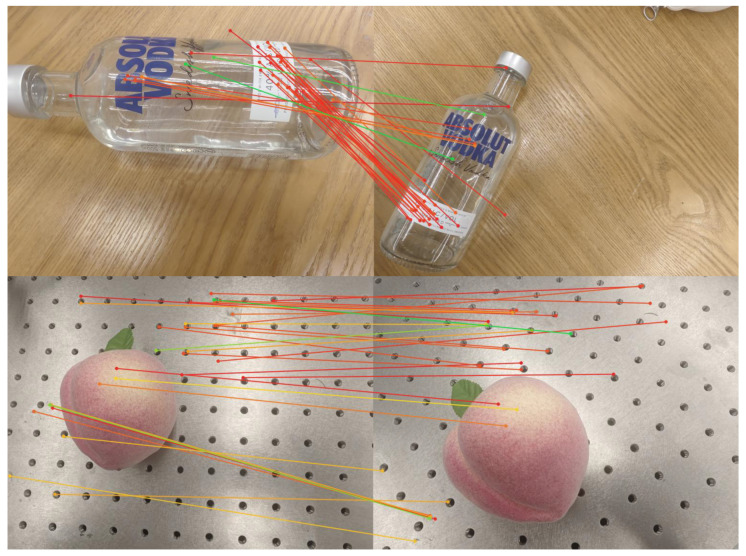
Representative failure cases of GAEFeat. Line colors indicate relative match confidence.

Figure 15 further illustrates representative failure cases of GAEFeat. For these examples, local features are first extracted using GAEFeat, followed by contextual matching between the image pairs via LightGlue. To retain as many candidate matches as possible, the matching threshold is set to 0, and the maximum number of keypoints is capped at 8000. The resulting matches are then sorted in descending order of confidence, and the top 30 are selected for visualization. The connecting lines are color-coded using a red-to-green gradient to denote the relative confidence within this Top-K subset, where red, yellow, and green indicate low, medium, and high confidence.

These examples show that the proposed model still has limitations when both local appearance and geometric verification become ambiguous. In the transparent bottle case, strong specular reflection, weak texture, and repeated logo-like patterns lead to visually plausible but physically incorrect correspondences. In the perforated-board case, repeated hole structures produce many locally similar descriptors, and some wrong correspondences can satisfy the estimated global geometry closely enough to be retained as false inliers. This indicates that although GAEFeat improves matching robustness in weakly textured scenes, local feature matching can still fail under severe reflection, repetitive structures, or insufficiently distinctive local geometry.

## 5. Discussion

Experimental results consistently demonstrate that the proposed geometry-aware design effectively improves matching robustness in weakly textured scenes. GAEFeat remains competitive on general scenarios such as MegaDepth-1500, indicating that introducing geometric constraints does not come at the cost of degrading baseline matching performance. As scene complexity increases, especially on ScanNet and our self-built industrial dataset, the performance advantage of the proposed method becomes more evident. This suggests that geometry-aware representation learning helps alleviate local geometric ambiguities and improves the model’s adaptability to near-field weakly textured environments.

Compared with lightweight front-ends that mainly rely on appearance cues or general-scene geometric priors [15,26,34], the key distinction of our method lies in the introduction of robot kinematics, hand–eye calibration, and RGB-D depth observations during training to automatically construct metric-scale cross-view supervision, surface-normal labels, and reliability weights. This design enables the network to learn more stable local geometric representations from near-field industrial scenes, while still relying only on RGB image input during inference, thereby balancing geometric robustness and deployment flexibility.

The ablation experiments further validate the effectiveness of the above designs. The geometric auxiliary branch guides the shared encoder to perceive local 3D surface structures through single-view normal supervision. The geometry-aware enhancement module further indicates that descriptor quality depends not only on representation learning under geometric constraints, but also on reliability-aware feature fusion during the aggregation stage. In other words, GAEFeat does not inject geometric information into the network in a direct or indiscriminate manner. Instead, it adopts a selective enhancement strategy, strengthening feature learning in regions with high geometric confidence while suppressing potentially misleading cues in regions affected by specular noise, depth holes, or unstable depth observations. This is particularly important for near-field industrial scenes, because the availability of depth data does not necessarily imply the reliability of geometric observations.

This design also reflects the difference between our method and depth-prior-assisted methods such as LiftFeat. LiftFeat uses large-scale monocular depth models to generate depth priors, which offers convenient data acquisition and can provide effective global structural information in general natural scenes. However, such predicted depth usually lacks strict metric-scale constraints, and its reliability may still be limited around object boundaries and near-field regions. Without an explicit reliability assessment mechanism, the model may have difficulty distinguishing effective geometric cues from misleading ones, which may introduce unstable supervision in near-field industrial scenarios. In contrast, our method further converts robot-derived metric-scale geometric information into a reliability-aware selective supervision strategy, improving matching robustness in weakly textured industrial scenes while maintaining compatibility with lightweight RGB-only inference.

This work does not aim for extreme lightweight design, but instead seeks to balance representation capability, robustness, and deployment efficiency within a lightweight network architecture. On standard general-purpose benchmarks, GAEFeat performs comparably to LiftFeat, but its computational cost is slightly increased due to the introduction of additional geometry-aware modules. It should be noted that this design is mainly intended to address weak textures, specular reflections, and geometric degradation in near-field industrial scenarios, with the goal of improving matching stability in complex industrial environments. During inference, the proposed framework still relies entirely on RGB images and internally predicted geometric cues, thereby maintaining good deployment flexibility. Experimental results show that GAEFeat achieves a GPU inference latency of only 3.9 ms on the NVIDIA Jetson AGX Orin platform. Although it is slightly slower than the most aggressively lightweight baseline models, it remains a practical visual front-end for edge-side robotic perception in industrial environments. This trade-off reflects the engineering-oriented design philosophy of this work.

Despite these advantages, the proposed model still has several limitations. The current supervision generation pipeline relies on a well-calibrated robotic acquisition system during training. This setup can provide supervision signals with metric-scale constraints, but when a robotic platform or accurate calibration is unavailable, its direct scalability will be limited. Nevertheless, the core idea of our framework is not restricted to robot-derived RGB-D geometric information, but lies in using reliable geometric cues to enhance feature representations in weakly textured regions. Therefore, other well-calibrated RGB-D acquisition systems, such as a turntable with a fixed rotation speed, can also serve as potential sources of geometric supervision. Meanwhile, some recent studies have explored combining high-precision measured depth with dense depth priors predicted by large models, which also provides a new direction for constructing more complete geometric supervision signals [51].

Beyond the scalability of supervision sources, the current method still leaves room for improvement in terms of depth degradation, appearance ambiguity, and system-level validation. In real near-field industrial scenarios, it remains difficult to rigorously and independently evaluate the specific influence of normal prediction quality on the final matching results. This difficulty is mainly caused by two factors. First, reliable pixel-level normal ground truth is difficult to obtain. Second, normal prediction quality usually affects the matching results indirectly through geometry-aware modulation and descriptor learning, rather than directly determining the final matching performance, which makes it difficult to design reasonable and effective normal perturbations. To further clarify the specific influence of normal quality on feature matching, systematic analysis under more controllable experimental conditions is required. This problem itself has considerable research value and can be regarded as an important direction for future work. Future studies may incorporate high-quality 3D scans, CAD-level geometric ground truth, or more accurate multi-view reconstruction results to further analyze the underlying mechanism by which variations in normal quality affect matching performance.

As shown in Figure 15, GAEFeat may still produce false inliers under severe specular reflection, transparent objects, or highly repetitive structures, indicating that appearance and geometric ambiguities have not been fully resolved. Introducing epipolar geometry constraints or lightweight post-processing strategies may further alleviate this issue. However, considering the lightweight design objective, the current version does not introduce additional explicit geometric verification mechanisms, but mainly relies on the implicit geometric representations learned by the model to reduce the influence of repetitive textures. Although the hybrid Sim-to-Real training strategy improves transferability, broader generalization to different sensors, materials, object categories, and real industrial environments remains an open problem. More real-world industrial datasets would further strengthen the conclusions, but such datasets remain limited due to acquisition cost, equipment requirements, privacy concerns, and deployment constraints. Future work will expand the evaluation to more diverse real industrial settings and RGB-D sensor configurations, while further exploring supervision signal construction, heterogeneous geometric prior fusion, normal prediction quality analysis, and explicit geometric constraints for repetitive-texture scenarios.

## 6. Conclusions

This paper presented GAEFeat, a lightweight geometry-aware feature matching framework tailored for weakly textured indoor industrial environments. By integrating robot kinematics, hand–eye calibration, and RGB-D observations, the framework constructs metric-scale cross-view correspondences, surface normals, and depth reliability cues as privileged training-time supervision, while preserving an efficient RGB-only inference mode during deployment.

The effectiveness of GAEFeat stems from three synergetic designs: robot-kinematics-guided supervision for scale-consistent labels, a surface-normal auxiliary branch for capturing 3D local morphology, and a reliability-aware enhancement module for adaptive visual-geometric fusion. Together, these components enhance the discriminability of lightweight descriptors under extreme viewpoint shifts, strong reflections, and severe texture degradation without adding inference-time overhead.

Extensive evaluations on public benchmarks (MegaDepth, ScanNet, HPatches, Aachen Day-Night v1.1) and a self-built industrial dataset demonstrate that GAEFeat delivers highly competitive performance, with particularly pronounced advantages in appearance-degraded near-field scenes. Furthermore, deployment on the NVIDIA Jetson AGX Orin platform yields a GPU inference latency of 3.9 ms, confirming its practical balance between matching robustness and edge-side computational efficiency as a visual front-end for robotic perception. Future work will investigate scalable supervision sources, broader generalization across diverse materials, and explicit verification mechanisms for highly repetitive structures.

## Figures and Tables

**Table 1:** Comparison of representative local feature matching methods in terms of design attributes, strengths, and limitations.

Category	Representative Methods	Main Design	Strengths	Limitations and Relevance to This Work
Handcrafted Features	ORB, SURF	Handcrafted keypoints and descriptors	Efficient and easy to deploy	Sensitive to weak texture, reflection, and viewpoint variation.
Learned Sparse Features	SuperPoint, D2-Net	Joint keypoint detection and descriptor learning	Stronger representation than handcrafted features	Mainly appearance-driven, with limited explicit 3D geometric constraints.
Context-Aware Matching	SuperGlue, LoFTR, ASpanFormer, DKM	GNN/Transformer-based or dense correspondence modeling	Robust under wide-baseline and large viewpoint changes	High computational and memory cost may limit edge deployment.
Lightweight Matching	LightGlue, XFeat, LIM	Adaptive matching or compact CNN design	Efficient on resource-limited devices	Limited modeling of industrial geometric priors.
Geometry-Aware Learning	LiftFeat	Geometric cues for local feature enhancement	Improves descriptor geometric consistency	Relies on geometric priors without explicit metric-scale constraints.
Geometry-Aware Learning	GAEFeat	Robot-kinematics-supervised geometry-aware learning	Robust under weak texture, reflective surfaces, and near-field viewpoint changes	Requires RGB-D, calibration, and robot poses for training data construction; maintains RGB-only inference.

**Table 2:** Structure of the self-built dataset.

Attribute	Simulation Data	Real Data
Platform/Sensor	Isaac Lab	UR5e + RealSense D435i
Dataset Name	Isaac-Home	InduReal-3D
Scene Type	Assembly workshop/household environment	Household items/industrial workpieces
Objects/Scenes	10 scenes	53 objects
Number of Samples	9972	2716
Number of Image Pairs	∼68 k	∼13 k
RGB Resolution	1280 × 720	1280 × 720
Depth Resolution	1280 × 720	1280 × 720
Depth Format	Float32	UInt16

**Table 3:** Training and inference usage of visual, geometric, and supervision signals.

Signal	Training	Inference	Role
RGB image $I_{t}$	Yes	Yes	Primary network input.
Depth map $D_{t}$	Yes	No	3D back-projection and geometric supervision construction.
Calibration and robot pose $K,T_{\mathrm{EC}},T_{\mathrm{BE}}\left(t\right)$	Yes	No	Metric camera pose estimation and cross-view reprojection.
Correspondence and consistency labels	Yes	No	Supervise keypoint detection and descriptor learning.
Normal labels and reliability maps	Yes	No	Guide the geometric auxiliary branch and reliability-aware enhancement.

**Table 4:** Dataset split statistics and acquisition conditions of the self-built hybrid dataset.

Attribute	Isaac-Home	InduReal-3D
Images	9972	2716
Training	7976 (80.0%)	2036 (75.0%)
Validation	998 (10.0%)	340 (12.5%)
Test	998 (10.0%)	340 (12.5%)
Split rule	Scene-level split	Category-level split
Material types	Household/workshop assets	Household objects/industrial workpieces
Lighting conditions	Controlled simulated lighting	Real indoor lighting
Sensor	Isaac Lab renderer	UR5e + RealSense D435i

**Table 5:** Relative pose estimation results. The upward arrow (↑) indicates that higher values are better.

Method	MegaDepth-1500 (AUC ↑)			ScanNet (AUC ↑)		
	5°	10°	20°	5°	10°	20°
ORB [9]	17.9	27.6	39.0	9.0	18.5	29.9
SuperPoint [11]	37.3	50.1	61.5	12.5	24.4	36.7
ALIKE [49]	40.5	56.9	68.2	9.8	19.5	30.3
SiLK [43]	39.9	55.1	66.9	15.9	30.1	44.5
XFeat [15]	42.6	56.4	67.7	16.7	32.6	47.8
LiftFeat [26]	**44.7**	**59.5**	**70.3**	18.5	34.9	51.2
Ours	43.8	59.1	69.4	**18.7**	**35.3**	**51.8**

Note: Bold indicates the best result in each column.

**Table 6:** Homography estimation results. The upward arrow (↑) indicates that higher values are better.

Method	Illumination (MHA ↑)			Viewpoint (MHA ↑)		
	3 px	5 px	7 px	3 px	5 px	7 px
ORB [9]	74.6	84.6	85.4	63.2	71.4	78.6
SuperPoint [11]	94.6	98.5	98.8	71.1	79.6	83.9
ALIKE [49]	94.6	98.5	**99.6**	68.2	77.5	81.4
SiLK [43]	78.5	82.3	83.8	48.6	59.6	62.5
XFeat [15]	95.0	98.1	98.8	68.6	81.1	86.1
LiftFeat [26]	**95.6**	**98.8**	99.2	71.1	81.7	87.5
Ours	**95.6**	98.5	99.1	**71.2**	**81.8**	**87.7**

Note: Bold indicates the best result in each column.

**Table 7:** Visual localization results.

Method	Day			Night		
	0.25 m/2°	0.5 m/5°	5 m/10°	0.25 m/2°	0.5 m/5°	5 m/10°
ORB [9]	66.9	76.1	93.7	10.2	12.2	19.4
SuperPoint [11]	87.4	**93.4**	97.0	77.6	85.7	95.9
ALIKE [49]	85.7	92.4	96.7	81.6	88.8	99.0
XFeat [15]	84.7	91.5	96.5	77.6	89.8	98.0
LiftFeat [26]	87.6	93.1	97.1	82.1	**89.9**	**99.1**
Ours	**87.8**	93.2	**97.2**	**82.3**	89.6	99.0

Note: Bold indicates the best result in each column.

**Table 8:** Relative pose estimation on the Isaac-Home simulated test split and the real-world InduReal-3D test split. The upward arrow (↑) indicates that higher values are better.

Method	Isaac-Home (AUC ↑)			InduReal-3D (AUC ↑)		
	5°	10°	20°	5°	10°	20°
ORB [9]	19.4	29.6	41.9	8.4	17.9	27.7
SuperPoint [11]	40.6	55.1	65.8	11.9	23.5	34.1
ALIKE [49]	44.1	61.3	75.4	9.5	18.6	28.3
SiLK [43]	41.8	60.7	72.1	14.9	27.9	42.7
XFeat [15]	47.7	65.6	78.9	16.4	32.0	46.3
LiftFeat [26]	48.2	66.9	79.2	17.9	34.6	48.1
Ours	**51.7**	**71.2**	**84.6**	**19.7**	**37.2**	**51.7**

Note: Bold indicates the best result in each column.

**Table 9:** Stepwise component ablation on the nighttime visual-localization test set.

Method	0.25 m/2°	0.5 m/5°	5 m/10°
Baseline backbone	78.8	85.9	97.5
+ Geometric auxiliary branch	79.9 (+1.1)	88.3 (+2.4)	98.4 (+0.9)
+ Geometry-aware enhancement w/o reliability gate	80.9 (+2.1)	88.7 (+2.8)	98.6 (+1.1)
+ Geometry-aware enhancement module	82.3 (+3.5)	89.6 (+3.7)	99.0 (+1.5)

**Table 10:** Comparison of computational cost on the embedded platform.

	SuperPoint	XFeat	LiftFeat	GAEFeat
Params (M)	1.30	0.66	0.85	0.92
FLOPs (G)	19.85	1.33	4.96	5.37
Descriptor Dim.	256	64	64	64
CPU Latency (ms)	133	19	25	27
GPU Latency (ms)	17.0	2.8	3.7	3.9

**Table 11:** Geometric auxiliary loss ablation on the InduReal-3D Test dataset.

$L_{\mathrm{kpt}}$	$L_{\mathrm{desc}}$	$L_{\mathrm{normal}}$	$L_{\mathrm{cv}}$	AUC@20°
✓	✓	–	–	44.5
✓	✓	✓	–	50.9 (+6.4)
✓	✓	✓	✓	51.7 (+7.2)

**Table 12:** Effect of Isaac-Home simulation scale and InduReal-3D real fine-tuning data on the InduReal-3D test set. The upward arrow (↑) indicates that higher values are better.

Isaac-Home Train		InduReal-3D Train	InduReal-3D Test (AUC ↑)		
Scenes	Images	Images	5°	10°	20°
0	0	0	16.6	29.7	44.1
0	0	2036	17.7	34.7	47.3
1	997	2036	17.9	34.8	46.4
2	1994	2036	18.3	35.3	48.3
3	2991	2036	18.6	35.7	49.2
4	3988	2036	19.1	36.4	50.1
5	4985	2036	19.2	36.6	50.7
6	5982	2036	19.5	37.0	51.2
7	6979	2036	19.6	37.1	51.5
8	7976	0	17.5	34.2	46.5
8	7976	2036	19.7	37.2	51.7

## Data Availability

The data presented in this study are available on request from the corresponding author. The data are not publicly available due to privacy and confidentiality restrictions associated with the specific industrial workshop scenes of our cooperative partners.
